# Investigating complementary and alternative medicine in Norwegian hospitals: a cross-sectional study with process evaluation

**DOI:** 10.1186/s12906-026-05339-w

**Published:** 2026-03-11

**Authors:** Trine Stub, Teklay T. Kidanemariam, Solveig Johansson, Agnete E. Kristoffersen

**Affiliations:** 1 Faculty of Health Science, Institute of Community Medicine, National Research Center of Complementary and Alternative Medicine (NAFKAM), UiT The Arctic University of Norway, Hansine Hansensveg 19, Tromsø, 9037 Norway; 2 Faculty of Health Science, Institute of Community Medicine, Master’s in Public Health, UiT The Arctic University of Norway, Hansine Hansensveg 19, Tromsø, 9037 Norway

**Keywords:** CAM, Complementary and Alternative Medicine, Hospitals, Norway

## Abstract

**Background:**

Hospitals worldwide have expanded their offerings of Complementary and Alternative Medicine (CAM) in recent years. However, there is a lack of updated data about CAM services offered in Norwegian hospitals. This study aimed to investigate the prevalence of CAM in Norwegian hospitals and explore how CAM is practiced within these hospitals.

**Methods:**

This study employed a mixed-methods approach, including a cross-sectional survey administered to all Norwegian hospitals (response rate, 74%; *n* = 215) and a process evaluation with healthcare providers (*n* = 11) practicing CAM at these hospitals. Survey data were analyzed using descriptive statistics, Pearson chi-square test, Fisher exact test, and binary logistic regression. Content analysis was employed to analyze the process evaluation data.

**Results:**

More than half of the hospitals (54%, *n* = 115) reported offering CAM to patients, and the highest prevalence was reported at clinics for substance abuse (77%, *n* = 27) and psychiatric hospitals (75% *n* = 41). Acupuncture (21%, *n* = 45), expressive art therapy (21%, *n* = 45), and yoga (21%, *n* = 45) were the most used modalities. In a process evaluation, four main themes were identified: the incorporation of CAM at hospitals, Reasons for offering CAM at hospitals, Advantages of offering CAM at hospitals, and the Safety of CAM.

**Conclusion:**

This study demonstrates that CAM is widely offered in Norwegian hospitals, consistent with previous research. While participants in the process evaluation viewed CAM as a holistic and beneficial approach to care, its adoption remains inconsistent across institutions. This variability, likely due to the lack of defined CAM roles and job descriptions, may pose indirect risks to both healthcare providers and patients. Standardized guidelines are needed to ensure safe and fair implementation.

**Supplementary Information:**

The online version contains supplementary material available at 10.1186/s12906-026-05339-w.

## Background

In Norway, visits to Complementary and Alternative Medicine (CAM) providers are prevalent and have been extensively studied across both healthy individuals [1–4] and those with specific health conditions [5–10]. These visits typically complement consultations with authorized healthcare providers [11]. In Norway CAM is defined as “…*health-related treatment that is performed outside the health and care service*,* and which is not performed by authorized health personnel or performed inside a public hospital or by authorized health personnel but with the methods that are largely used outside a public hospital*” [12]. Massage therapy, acupuncture, and healing are CAM modalities most frequently utilized. Over the past decade, the prevalence of visits to CAM providers decreased from 37% in 2012 [1] to 21% in 2020 [1], followed by a gradual increase to 26% in 2024 [13].

The use of CAM in Norwegian hospitals has been examined in three studies conducted in 2001 [14], 2008 [15], and 2013 [16]. These studies demonstrate a significant rise in the proportion of hospitals that offer CAM, from 25% in 2001 [14], to 64% in 2013 [16]. The 2013 study highlighted that CAM services were available in public and private Norwegian hospitals, encompassing somatic and psychiatric facilities. Notably, the most substantial increase in CAM utilization occurred in psychiatric hospitals, where the provision rose from 29% in 2008 [15] to 77% in 2013 [16].

Acupuncture has emerged as the most frequently utilized CAM modality in Norwegian hospitals. Its usage increased significantly from 8.0% in 2001 [14], to 40% in 2008 [15], followed by a slight decline to 37.% in 2013 [16]. Similarly, the use of massage therapy rose from less than 1% in 2001 to 15% in 2013. Expressive arts therapy also saw a notable increase, from approximately 1% in 2001 [14], to 25% in 2013 [16]. Moreover, the within-hospital practice of CAM in Norwegian hospitals often depended on one person who actively provided CAM to their patients [17].

A Danish study [15] conducted in 2008, revealed that 33% of somatic hospitals and 23.% of psychiatric hospitals offered CAM, with a difference regarding these services between public (34%) and private (21%) institutions. Nearly all hospitals offered acupuncture while only one hospital offered Eye Movement Desensitization and Reprocessing (EMDR) therapy and Light Therapy [15].

Wemrell et al. found in a Swedish study that CAM was available in 62% of the 489 responding psychiatric units, primarily for managing symptoms such as anxiety, sleep disturbances, and depression [18]. The main motivations for offering CAM included symptom relief, responding to patient requests, and reducing pharmaceutical medication use [18]. The top five modalities offered were mindfulness (43%), basal body awareness (28%), massage or tactile stimulation (23%), acupuncture (19%), yoga, and Tai Chi or Qigong (15%).

In Europe, CAM modalities are practiced both in conventional healthcare settings by medical providers and outside these facilities by non-medical providers [19]. The prevalence of CAM use was 18% in 2020, including acupuncture, acupressure, chiropractic, osteopathy, and reflexology [20]. Data suggest that roughly 305,000 CAM providers practice within the EU, of whom approximately 160,000 are non-medical and 145,000 are medical providers.

Research has demonstrated variations in attitudes towards CAM among different healthcare providers in hospitals. Attitudes towards CAM are shaped by a variety of factors, including personal beliefs and experiences [21], lack of scientific evidence [22, 23], safety considerations [24, 25], and healthcare providers’ dedication to respect patients’ choices and needs [21]. However, research indicates that attitudes towards CAM vary among different healthcare providers in hospitals. For instance, a related Norwegian study [25], which examined the attitudes and knowledge of conventional and CAM healthcare providers in cancer care, found that most of the medical doctors (61%) and nurses (55%) would have neither discouraged nor encouraged the use of CAM modalities if patients asked them for advice. It was also found that 94% of medical doctors and 93% of nurses voiced concerns about the risks associated with CAM modalities.

A study from New Zealand [26] highlighted that healthcare providers generally held a positive view toward patients’ use of CAM. However, they expressed concerns regarding the lack of scientific evidence supporting many CAM modalities. They were also concerned about issues regarding regulation, safety, and financial costs. Consequently, they advocated for an evidence-based approach to CAM practice and called for stronger regulatory measures. These findings underscore the need for enhanced education of conventional healthcare providers to ensure the safe and effective adaptation of CAM into the conventional healthcare system.

The use of CAM offered at Norwegian hospitals has previously been examined [14–16]. However, to describe and update these data this study aimed to.

1. investigate the prevalence of CAM modalities offered at Norwegian hospitals (in a cross-sectional survey), and.
2. explore how CAM is practiced among the employed healthcare providers at these hospitals (in a process evaluation).

## Methods

This research project compromises a cross-sectional study among Norwegian hospitals with a process evaluation among healthcare providers with additional training in CAM employed at these institutions.

### Setting

Norway follows the Nordic health model of universal healthcare [27], which is designed to provide comprehensive health services to all residents. It is primarily financed through taxes and managed by the Ministry of Health and Care Services. The Norwegian government funds the official hospitals which are managed by four Regional Health Authorities (RHAs). Healthcare is organized into three levels: primary, specialist, and municipal health services [27]. While the public system covers most services, a small private sector offers healthcare services to the inhabitants, and several have funding contracts with the four RHAs.

### Inclusion and exclusion criteria

*The inclusion criteria* comprise all official and private hospitals in Norway that provide health care to patients, and private hospitals with a funding agreement with RHAs.

*The exclusion criteria* comprise private hospitals without a funding contract with the RHA. This exclusion was made to ensure the selection of a representative sample of healthcare facilities that are accessible to the broader Norwegian population, including patients with diverse socioeconomic backgrounds.

### Recruitment and data collection

Initially, 290 hospitals and institutions were identified for the study. A research advisor from UiT, the Arctic University of Norway, contacted all hospitals to identify a senior medical officer willing to complete the questionnaire on behalf of the organization. This was successfully established in 286 hospitals. The questionnaire, accompanied by a cover letter and a list of the 43 most commonly used CAM modalities in Norway, was distributed to these contacts (*n* = 286) in October 2019.

**Fig. 1:**
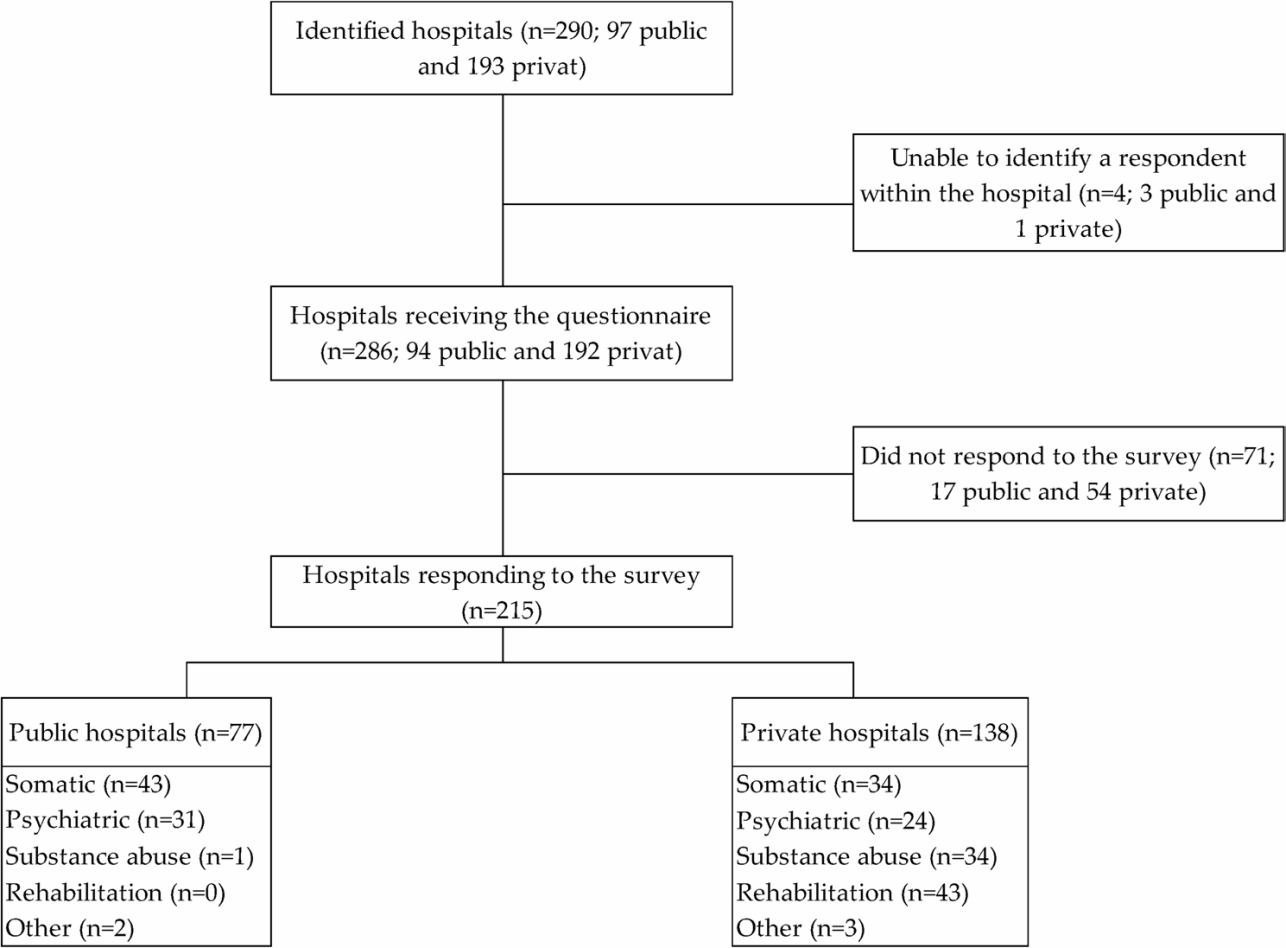
Flow chart of participating hospitals

Of the 286 hospitals that received the questionnaire, 215 responded, resulting in a response rate of 74%. The respondents comprised 77 public and 138 private hospitals. These were further categorized into somatic (*n* = 77), psychiatric (*n* = 55), substance abuse (*n* = 35), rehabilitation (*n* = 43), and other types of hospitals (*n* = 5). Notably, public hospitals exhibited a higher response rate of 81.9% compared to 71.8% for private hospitals (see Fig. 1).

### The cross-sectional study

#### The questionnaire

The survey questionnaire was adapted from the 2013 hospital study [16]. Participants were asked if the following modalities were offered at their hospital, with the following response alternatives: *acupuncture*, *massage*, *psychotherapy (excluding services by psychologists/psychiatrists)*, *expressive arts therapy* (including visual arts and music therapy), *diet recommendations*, and/or *other CAM modalities* with an option for the response of *no CAM modalities offered*. Respondents were also asked to provide contact information for healthcare providers offering CAM at the hospitals, as these providers would later be invited to participate in a process evaluation. The questionnaire was only one page.

### Statistics/ power calculation

To achieve adequate study power, a minimum sample size of *n* = 166 was calculated based on a desired margin of error of 5%, a confidence level of 95%, and an assumed heterogeneity of 50%. This sample size was necessary to represent the 290 identified Norwegian hospitals [28]. The sample size of 215 hospitals, was considered sufficient for this purpose. Descriptive statistics were performed using cross-tabulation and frequency analyses. For between-group analyses, Pearson’s chi-square tests and Fisher’s exact tests were applied to categorical variables, while independent sample t-tests were used for continuous variables. Binary logistic regression was used for adjusted values. Statistical significance was determined at a level of (*p* < 0.05). All statistical analyses were conducted using the Statistical Package for the Social Sciences (SPSS) version 29.0.

### Process evaluation

In a process evaluation, 11 healthcare providers working at the hospitals were interviewed and asked to reflect on their CAM practice. The aim was to gather information about how CAM was practiced at the hospitals. The interviews took place from June through August 2023.

### The interviews

The interviews were semi-structured, allowing the participants to express their views in their own words [29]. The interviews were based on an interview guide established on an earlier study [14], scientific literature, and the knowledge of the research team. The participants were asked to reflect on *how CAM is practiced at the hospital; the incorporation process; the advantages of CAM; collaboration with colleagues and possible adverse effects of CAM.* In addition, demographic data; training (conventional and CAM); and years in clinical practice were collected.

### The participants

In the questionnaire the contact persons at each hospital were asked to provide contact information to healthcare providers who offered CAM at the hospitals. They were contacted by the research assistant and asked to participate. From a list of 40 healthcare providers, 12 accepted the invitation. However, one participant withdrew, and a total of 11 providers with training in CAM were included in this evaluation.

The participants were all females. Their mean age was 53 years and the mean years in CAM practice was 16 years. At the time of the interview, all participants were employed as conventional healthcare providers and practiced CAM either as a sole modality or in combination with conventional medicine at the hospitals (Table 1: demographic data of the participants).

**Table 1:** Demographic data of the participants

Gender	Females (*n* = 11)
Age	53 years (mean)
	34–67 years (range)
Training in conventional medicine	Nurse (*n* = 3)
	Physiotherapy (*n* = 3)
	Sociology (*n* = 1)
	Child welfare nurse (*n* = 1)
	Psychology (*n* = 1)
Training in CAM**	Art therapy (*n* = 4)
	Acupuncture (*n* = 3)
	Mindfulness/meditation (*n* = 2)
	Music therapy (*n* = 2)
	Eye movement desensitization and reprocessing (EMDR) therapy (*n* = 1)
	Yoga (*n* = 1)
Years in conventional practice	18.5 years (mean)
	12–40 years (range)
Years in CAM practice	16 years (mean)
	4–30 years (range)
Workplace	BUP (*n* = 1)*
	BUPA (*n* = 2)**
	DPS (*n* = 7)***
	Pain Clinic (*n* = 1) ****

*BUP Child and Adolescent Psychiatric outpatient clinic, **BUPA Child and Adolescent Psychiatric clinic, ***DPS District psychiatric center, **** Pain clinic at a somatic hospital

### Data collection

Eleven participants were interviewed individually. The interviews were audio-recorded and transcribed *ad verbatim* [30]. The interviews were conducted in Norwegian and translated into English by the second author (TT). The interviews were facilitated by one researcher (TT). He was a master’s student in public health when the data was collected, and this study forms the basis of his master thesis. He did not know the participants before the interviews. The interviews were conducted by telephone. The interviews lasted between 30 and 60 min and notes were taken during the interviews.

### Data analysis

The data analysis was conducted according to conventional content analysis [31] which involves systematically categorizing and interpreting data to identify patterns and meanings that emerge during analysis. The first (TS) and second author (TT) read the transcripts several times. The data were organized according to the aim of the process evaluation (how CAM is practiced in Norwegian hospitals) and categorized into relevant themes and codes. Finally, due to identical information, codes and themes were collapsed into 4 main themes (incorporation of CAM at the hospitals; reasons for offering CAM; advantages of offering CAM, and Safety of CAM), see Table 2. Nvivo 1.6154 was used for data management and analysis [32].

**Table 2:** Overview of the main themes and sub-themes

Themes	Sub-themes
Incorporation of CAM at hospitals	-*Participant’s interests*
	*-A manager with interests and beneficial experience in using CAM*
	*-Dissociation from the CAM field*
Reasons for offering CAM at hospitals	-*People’s needs are different*
	*-Conventional therapies do not work for everyone*
Advantages of offering CAM at hospitals	*-Access to emotions in a different way than conventional treatment*
	*- a holistic approach to care*
	*-Provide individuals with a strategy they can use later in life*
Safety of CAM	

## Results

### Results of the survey

#### Basic characteristics of the surveyed hospitals

The majority of participating hospitals (54%, *n* = 116) were located in the southeastern region of Norway (*Helse Sør-Øst*), followed by the western region (*Helse Vest*, 20 %, *n* = 42), the northern region (*Helse Nord*, 14%, *n* = 30), and the central region (*Helse Midt-Norge*, 13%, *n* = 27). Most hospitals were private (64%, *n* = 138), with the highest concentration of private hospitals in the southeastern region (76%, *n* = 88, *p* < 0.001). Across all regions, somatic hospitals were the most common type (36%, *n* = 77), followed by psychiatric hospitals (26%, *n* = 55), rehabilitation facilities (20%, *n* = 43), and substance abuse centers (16%, *n* = 35). There were some regional variations in these distributions (*p* = 0.034) (see Table 3).

**Table 3:** Basic characteristics of the hospitals included

	Total	Southeastern Norway (Helse Sør-Øst)	Western Norway (Helse Vest)	Central Norway (Helse Midt-Norge)	Northern Norway (Helse Nord)	*p*-value
	*n* (%)	*n* (%)	*n* (%)	*n* (%)	*n* (%)	
Somatic	77 (35.8)	34 (29.3)	17 (40.5)	13 (48.1)	13 (43.3)	0.034^
Psychiatric	55 (25.6)	28 (24.1)	16 (38.1)	2 (7.4)	9 (30.0)	
Substance abuse	35 (16.3)	26 (22.4)	4 (9.4)	3 (11.1)	2 (6.7)	
Rehabilitation	43 (20.0)	24 (20.7)	5 (11.9)	9 (33.3)	5 (16.7)	
Other	5 (2.3)	4 (3.4)	0 (0.0)	0 (0.0)	1 (3.3)	
Public	77 (35.8)	28 (24.1)	21 (50.0)	11 (40.7)	17 (56.7)	< 0.001*
Private	138 (64.2)	88 (75.9)	21 (50.0)	16 (59.3()	13 (43.3)	
**Total**	**215 (100)**	**116 (54.0)**	**42 (19.5)**	**27 (12.6)**	**30 (14.0)**	

^Fisher exact test

* Pearson chi-square test

#### Prevalence of CAM use

Of the 215 hospitals surveyed, 54% (*n* = 115) reported offering CAM to their patients. The most commonly offered CAM modalities were acupuncture, expressive arts therapy, and yoga, each available at 21% of the hospitals. These were followed by meditation/mindfulness (15%), massage (8%), and psychotherapy services not provided by psychologists or psychiatrists (4%) (see Table 3).

Hospitals specializing in substance abuse were the most likely to offer CAM (77%, *n* = 27), followed by psychiatric hospitals (75%, *n* = 41), rehabilitation hospitals (48%, *n* = 20), and somatic hospitals (34%, *n* = 26). These differences were statistically significant (*p* < 0.001) (Table 4).

**Table 4:** CAM modalities offered across different hospitals

	Total	Somatic	Psychiatric	Substance abuse	Rehabilitation	Other	
	*n* (%)	*n* (%)	*n* (%)	*n* (%)	*n* (%)	*n* (%)	*p*-value^
Acupuncture	45 (20.9)	18 (23.4)	5 (9.1)	18 (51.4)	4 (9.3)	0 (0.0)	< 0.001
Expressive arts therapy	45 (20.9)	4 (5.2)	30 (54.5)	8 (22.9)	3 (7.0)	0 (0.0)	< 0.001
Yoga	45 (20.9)	4 (5.2)	16 (29.1)	13 (37.1)	11 (25.6)	1 (20.0)	< 0.001
Meditation/mindfulness	32 (14.9)	2 (2.6)	10 (18.2)	10 (28.6)	10 (23.3)	0 (0.0)	< 0.001
Massage	17 (7.9)	7 (9.1)	1 (1.8)	7 (20.0)	2 (4.7)	0 (0.0)	0.039
Psychotherapy	9 (4.2)	1 (1.3)	2 (3.6)	5 (14.3)	1 (2.3)	0 (0.0)	0.051
Alternative diet^2^	7 (3.3)	2 (2.6)	0 (0.0)	3 (8.6)	2 (4.7)	0 (0.0)	0.202
Gestalt therapy	3 (1.4)	0 (0.0)	0 (0.0)	3 (8.6)	0 (0.0)	0 (0.0)	0.009
Tai chi / qi gong	3 (1.4)	0 (0.0)	0 (0.0)	0 (0.0)	3 (7.0)	0 (0.0)	0.040
Aromatherapy	2 (1.0)	0 (0.0)	1 (1.8)	1 (2.9)	0 (0.0)	0 (0.0)	0.328
Other^1^	6 (2.8)	1 (1.3)	0 (0.0)	4 (11.4)	1 (2.3)	0 (0.0)	0.035
**Total**	**115 (53.5)**	**26 (33.8)**	**41 (74.5)**	**27 (77.1)**	**20 (47.6)**	**1 (20.0)**	**< 0.001**

^1^ *EMDR* Eye Movement Desensitization and Reprocessing (*n* = 1), osteopathy (*n* = 1), reflexology (*n* = 1), thought field therapy (*n* = 1), receptive sound therapy (*n* = 1), and unidentified (*n* = 1)

^2^Offered at hospitals to improve health

^Fisher exact test

Somatic hospitals were most likely to offer acupuncture, with 23% (*n* = 18) providing this service. In contrast, psychiatric hospitals predominantly offered expressive arts therapy, with 55% (*n* = 30) including it in their services. Institutions specializing in substance abuse treatment and rehabilitation were most likely to offer yoga, with rates of 23% and 26%, respectively (*p* < 0.001). Meditation and mindfulness were less commonly used in somatic hospitals but were frequently offered in psychiatric, substance abuse, and rehabilitation settings (18%, 29%, and 23%) respectively (*p* < 0.001). Notably, institutions that focused on substance abuse treatment were the most likely to offer massage, with 20% providing this service (*p* = 0.039).

Hospitals in the southeastern and northern regions were most likely to offer CAM, with 61% and 57% of hospitals doing so, respectively. In contrast, hospitals in central Norway were the least likely to offer CAM to their patients (33%, *p* = 0.042) (see Table 5). Hospitals in the central and northern regions were most likely to offer acupuncture, with rates of 15% and 37%, respectively (*p* = 0.016). In the southeastern region, hospitals were most likely to offer yoga (30%, *p* = 0.003). Additionally, hospitals in the western region were the most likely to provide expressive arts therapy (31% *p* = 0.029) (see Table 5).

When adjusting for the type of hospital (somatic, psychiatric, substance abuse, and rehabilitation), regional differences remained statistically significant only for yoga (*p* = 0.002) and art therapy (*p* = 0.047) (see Table 5).

**Table 5:** Regional disparities of hospitals offering CAM in Norway

CAM	Total	Southeastern Norway (Helse Sør-Øst)	Western Norway (Helse Vest)	Central Norway (Helse Midt-Norge)	Northern Norway (Helse Nord)		
	*n* (%)	*n* (%)	*n* (%)	*n* (%)	*n* (%)	*p*-value	Adjusted *p*-value for type of hospital’’
Acupuncture	45 (20.9)	27 (23.3)	3 (7.1)	4 (14.8)	11 (36.7)	0.016*	0.479`
Expressive arts therapy	45 (20.9)	27 (23.3)	13 (31.0)	2 (7.4)	3 (10.0)	0.029*	0.047`
Yoga	45 (20.9)	35 (30.2)	6 (14.3)	2 (7.4)	2 (6.7)	0.003*	0.002`
Meditation/ mindfulness	32 (14.9)	20 (17.2)	4 (9.5)	2 (7.4)	6 (20.0)	0.384^	0.998`
Massage	17 (7.9)	12 (10.3)	0 (0.0)	1 (3.7)	4 (13.3)	0.031^	0.857`
Psychotherapy	9 (4.2)	6 (5.2)	2 (4.8)	0 (0.0)	1 (3.3)	0.076^	0.446`
Alternative diet	7 (3.3)	7 (6.0)	0 (0.0)	0 (0.0)	0 (0.0)	0.189^	0.995`
Gestalt therapy	3 (1.4)	3 (2.6)	0 (0.0)	0 (0.0)	0 (0.0)	0.827^	0.995`
Thai-chi / Qi gong	3 (1.4)	1 (0.9)	0 (0.0)	1 (3.7)	1 (3.3)	0.216^	0.180`
Aromatherapy	2 (1.0)	2 (1.7)	0 (0.0)	0 (0.0)	0 (0.0)	1.000^	0.995`
Other^1^	6 (2.8)	5 (4.3)	0 (0.0)	0 (0.0)	1 (3.3)	0.554^	0.476`
**Total**	**115 (53.5)**	**70 (60.9)**	**19 (45.2)**	**9 (33.3)**	**17 (56.7)**	0.042*	**0.181`**

^1^EMDR (*n* = 1), osteopathy (*n* = 1), reflexology (*n* = 1), thought field therapy (*n* = 1), receptive sound therapy (*n* = 1), and unidentified (*n* = 1)

^Fisher exact test

*Pearson chi square test

`Binary logistic regression

‘’somatic, psychiatric, substance abuse and rehabilitation

### Results of the process evaluation

In a process evaluation, participants were asked to reflect on *their CAM clinical practice* and how the *modality was integrated* at the ward, as well as the *advantages of CAM*, *collaboration with colleagues*,* and possible adverse effects of CAM*, which are presented below.

#### Incorporation of CAM at hospitals

Several key factors informed the incorporation of CAM, such as adherence to national guidelines [33, 34], its designation as a drug-free treatment option [35], the availability of healthcare providers trained in the specific modality (except for the music therapist, who were trained in learning science in addition to music therapy), and formal approval from a department manager. These treatments were administered during regular working hours as an integral component of conventional care.

#### Participants’ interest

Most participants were employed as conventional healthcare providers but pursued CAM due to personal interest and training in CAM. Their motivation to implement CAM at the hospital stemmed from practical experiences with CAM.

The participants actively informed their colleagues about the benefits of CAM. They advocated for the modality in department meetings where the staff planned patient treatment protocols. They invested considerable time in gathering evidence-based information about the modality and argued that CAM was equally important as conventional treatments offered at the hospitals.

Participant (ID4) observed that there was a perception among her colleagues that music therapy was pleasant and enjoyable for the patients, but if there was a scheduling conflict with a doctor or psychologist, the latter was prioritized. However, over the years she has made efforts to work professionally and concretely to integrate music therapy into the overall treatment process. *Today I feel that music therapy is regarded equally as important as conventional treatments that are offered at the ward*, she said.

In line with this, participant (ID6) said that she was busy telling her colleagues about expressive art therapy when she started. She attended various meetings where she informed them about the modality. In this way, they got to know her and her competence.

Participant (ID5), a physiotherapist and expressive art therapist, said that they had specialists in cognitive therapy and trauma therapy at the ward, and she was the specialist in using creative expression methods and the body, which the others did not know so much about. However, she said: *The colleagues here complement and help each other.*

Participant (ID10) held a specialist position at the hospital, which means she had independent responsibility for her patients. This gave her considerable freedom in shaping her work. She worked interdisciplinarily with doctors, psychologists, and cognitive therapists.

#### A manager with interest and beneficial experience in using CAM

Two participants (ID11, ID5) experienced that their manager supported CAM implementation, drawing from positive experiences in the past working in hospitals where CAM was implemented and offered to patients.

Working at a pain clinic, the participant (ID11) described the introduction of acupuncture. She said that the decision to implement this modality was made by the clinic’s manager who had prior experience working at a regional hospital where acupuncture was implemented as a treatment. Subsequently, the manager recruited her (ID11) for the physiotherapy and acupuncturist position. She stated: *It was the manager who wanted an acupuncturist in the position. Then I was sort of headhunted. I was asked if I would like to work as an acupuncturist at the clinic.*

Participant (ID5) experienced that her manager had other motives to implement CAM as she noted: *The chief psychologist at the hospital had a different (alternative) mindset in terms of health*,* so she hired me.*

In contrast, participant (ID4) experienced a different approach when deciding on modalities to offer patients at the hospital. The management consulted evidence-based guidelines when planning a new department for substance abuse treatment (TSB). The guidelines recommended music therapy as an evidence-based modality for substance abuse. Following this recommendation, the institution decided to hire a music therapist in 2018, leading to the *recruitment for the position.*

#### Disassociated from the CAM field

According to Norwegian law, these modalities are classified as CAM. However, six participants (ID3, ID4, ID9, ID5, ID6, ID8) expressed frustration about being associated with the CAM field, because many individuals and conventional healthcare providers perceive CAM as lacking scientific evidence for effects. (ID6) said: *It’s sad that we are classified as CAM because this modality (art therapy) is clearly rooted in the same theories as other psychotherapeutic approaches.*

Although modalities such as mindfulness, art therapy, and music therapy are supported by evidence, these participants deliberately distance themselves from the CAM label, illustrated by (ID3): *We don’t think of music therapy as CAM. Music therapy is evidence-based*,* has proven effects*,* and is actually recommended by the health authorities in the national guidelines.*

Moreover, (ID8) expressed: *There is nothing related to quackery or mysticism about it (art therapy) whatsoever.*

None of the participants had formal job descriptions for their roles related to CAM. According to participant (ID11), the reason is that there is *no established employment arrangement for CAM positions in the official health care system in Norway*, she said.

#### Reasons for offering CAM at hospitals

Medical treatment is the first treatment option for patients in psychiatric hospitals in Norway. Treatment usually combines medication and psychotherapy in multidisciplinary, patient-tailored care. Medication is common for severe disorders (psychosis, bipolar disorders), while psychotherapy, especially Cognitive Behavioral Therapy (CBT), is central for depression, anxiety, and eating disorders. Acute inpatient care is used when 24/7 support is required [36].

However, the participants noted several reasons for implementing CAM at the hospitals, all related to different patients’ health needs.

#### People’s needs are different

Participants emphasized that patients have varied needs, and a one-size-fits-all approach is insufficient. For instance, music therapy emerged as a powerful tool for fostering self-understanding and engagement. Participant (ID3) observed that many individuals in the community lack the motivation to engage in traditional talk therapy but show enthusiasm for musical activities. This modality provides an alternative pathway for patients to explore their emotions and experiences.

Similarly, Participant (ID7) shared how she helps young patients rehearse strategies for managing distressing situations, such as attending a funeral where an assailant might be present. By practicing scenarios—like deciding where to sit in the church to avoid contact—patients feel more prepared and confident. This hands-on approach was noted to be more effective than merely discussing the situation.

Physiotherapy also plays a significant role in addressing physical and emotional needs. A physiotherapist (ID2) who extensively used acupuncture for pain reduction in patients undergoing rehabilitation following surgery said: *I always apply physiotherapy to my patients*,* as physiotherapy is my primary occupation and position*. *I use acupuncture quite often*,* but many of the points I use as an acupuncturist also correspond to trigger points in physiotherapy treatment*.

Participant (ID5) highlighted the importance of offering diverse modalities, likening it to trying different medications: *I think it’s really important to offer many different modalities. Just like trying different medications*,* if one drug doesn’t work*,* you can try another. I believe that since people and patients are different*,* there are different ways to help them.*

#### Conventional therapies do not work for everyone

Participants experienced that conventional therapies, such as talk therapy, are not effective for everyone. Children, for example, often struggle to express their emotions verbally. Participant (ID7) emphasized the need for therapists to adapt their approaches to suit each child’s needs, noting that expressive arts and play therapy are more natural and effective for younger patients. *Art is such a natural language for children*, she explained, *making it easier to connect with them through play and artistic expression*.

For patients with psychosis, verbal communication can be particularly challenging. Participant (ID3) described how music therapy provides empowering experiences and strengthens patients’ resources, serving as a foundation for further therapeutic work. She noted that patients with psychosis often lose track of conversations, making non-verbal modalities like music therapy invaluable.

Art therapy also emerged as a key alternative. Participant (ID5) explained, *Art therapy is a good option because it involves the body*,* the senses*,* and creativity—not just the head.* Participant (ID6) added that art therapy can *awaken the senses* in patients whose bodies have become numb or passive, offering a vital alternative to cognitive treatments.

Norway’s multicultural society presents additional challenges for conventional therapies. Participant (ID6) shared her experience working with women from multicultural backgrounds who struggled to express feelings of depression or anxiety verbally, especially when an interpreter was present. The presence of a stranger in the room often felt intimidating, making talk therapy less effective. Expressive and art activities allowed patients to express themselves in ways that felt natural and empowering, bypassing the barriers of language and cultural differences. CAM approaches, such as expressive arts therapy and acupuncture, provide valuable alternatives that address the unique needs of patients, fostering engagement, empowerment, and healing.

#### Advantages of offering CAM at hospitals

Offering CAM at hospitals allows patients to seamlessly access conventional and CAM modalities without the logistical challenges of coordinating care across separate facilities. The hospital setting fosters better communication and collaboration among healthcare providers, who can easily share patient information within the same system. In contrast, cooperating with CAM providers outside hospitals is often more challenging, and finding CAM providers trained to treat specific patient groups can be difficult for hospital-based providers.

Participants reported that CAM enables patients to engage with their emotions in ways that differ from conventional treatments. CAM was perceived as a holistic and beneficial approach, as it often fosters an enhanced sense of mastery, confidence, and self-awareness among patients. Additionally, these modalities provide individuals with strategies that can be applied throughout their lives.

#### A holistic approach to care

The participants believed that CAM is a holistic and beneficial approach that involves the body, the senses, and creativity. One of the advantages of music therapy is that it fosters a sense of achievement, potentially boosting self-confidence and helping individuals feel more secure in themselves. For instance, patients who have never played the guitar before may find themselves performing on stage in front of 50 others at the institution. After accomplishing such a task, the positive feeling of working on something, mastering it, and succeeding was crucial. It helps individuals to gain the confidence needed to manage their lives moving forward, emphasized (ID4). In line with this, participants (ID3) believed that music therapy provides a comprehensive and holistic view of patient care.

Moreover, participant (ID5) believes that reflecting on the process after a session may give the patients new perspectives. She continued: *The advantage of art therapy is that it is holistic. There are a lot of people who can’t access their emotions. Emotions are like an entirely unknown landscape for them. Creative methods and bodywork can make it easier for them to access their emotions and inner life.*

Participant (ID6) asked a group of young people what they had learned through 14 weeks of art therapy. They replied that they were unaware they had so many resources, skills, interests, and knowledge. *This was good to hear*, she said.

#### Access to emotions in a different way than conventional treatment

Participant (ID9) believed that mindfulness is a well-documented practice and a better approach than medical treatment for many patients at the clinic. She stated that *the patients should get out of what we call autopilot and try to make better choices instead of using drugs. Through mindfulness exercises*,* the idea is to face the discomfort rather than run away from feelings (using drugs).* Another participant (ID1) explained that many of her patients suffer from stress and sleep issues, leading to exhaustion. She noted that mindfulness helped them calm down their nervous systems. Additionally, it improves their mental focus, and they *become more receptive to conventional treatments.* She often combines mindfulness with CBT to enhance patients’ self- and body awareness.

#### Provide individuals with a strategy they can use later in life

Participant (ID4) emphasized the importance of providing patients with positive coping experiences (empowering). She observes that patients who have participated in music therapy have an activity they can use after returning home. *They can*,* for instance*,* join a choir or play music weekly with others as part of an aftercare program*, she noted.* This may also create a new social network, leading to an improved circle of friends*, she continued.

#### Safety of CAM

All participants in the study indicated that CAM was safe. However, they highlighted several strategies employed within CAM practices to minimize risk and ensure patient safety. They also emphasized the importance of taking precautions to ensure modalities were positive and connected with few risks. They also emphasized the responsibility of the providers to take caution and guide the patients carefully during therapy, which requires training and certification, as described below.

Participant (ID1) mentioned consulting with colleagues before administering therapy to identify any red flag issues. Another participant (ID2) emphasized the importance of anatomical knowledge and skill in acupuncture to ensure safety, stating that *one should know the anatomy of the body to place the needles correctly*.

She also advocated for patient autonomy, stressing the importance of providing sufficient information to patients and involving them actively in the decision-making and treatment process. She clarified that she never imposed demands on patients and respected their choices.

Participant (ID8) described EMDR therapy as generally safe but noted that it is only administered after obtaining approval from a specialist. Similarly, another participant (ID9) characterized mindfulness as a painless approach with no identified adverse effects, although she acknowledged that some individuals might find aspects of the therapy uncomfortable due to unfamiliarity with the practice of sitting and noticing their mindful experiences. She said, *however*,* that this is not regarded as traditional adverse effects.*

According to participant (ID10), expressive arts therapy can sometimes bring latent feelings to the surface, which may be challenging for patients. She emphasized the importance of person-to-person interactions in supporting individuals facing life challenges and stressed that CAM providers should be trained to understand their limitations and be sensitive to not pushing patients beyond their comfort zones, which she regarded as risky.

For patients with a history of addiction, music can significantly impact emotions and behaviors. In administering music therapy, the participants (ID3, ID4) suggested that therapists need to be aware of music’s potential to trigger emotions and cravings, such as a desire for drugs. They stressed that *in cases where patients have a history of using drugs when listening to music*,* the music can be a powerful trigger*.

To minimize harm to patients, a participant (ID10) added: *I believe it is important not to use acupuncture for conditions where there is evidence supporting better outcomes with other treatments. Therefore*,* I think one should be very selective in its application.*

In some hospitals, wearing alarms was considered an essential part of patient care and safety protocols. Participant (ID4) clarified that she carries an alarm for her safety but has never activated it during work hours. She emphasized that *although she hasn’t used the alarm*,* its presence reflects a commitment to creating a secure environment for both her and her patients.*

## Discussion

This study found that more than half of the hospitals in Norway offered CAM to their patients. The modalities most used were acupuncture, expressive arts therapy, and yoga, and they were commonly offered at substance abuse clinics and psychiatric hospitals. Employees’ interests drove the incorporation of CAM into hospitals. They actively promoted CAM in departmental meetings and emphasized the need for diverse treatment options to address unique patient needs, as conventional medicine did not work for everyone. CAM was described as a holistic approach that enhances emotional engagement, mastery, confidence, and self-awareness. Participants affirmed CAM’s safety and stressed the importance of provider responsibility, highlighting the need for proper training and certification. However, participants lacked formal job descriptions due to the absence of established CAM roles in Norway’s healthcare system.

### Other studies

In 1997, only six Norwegian hospitals offered CAM [37]. Later, the prevalence of CAM offered in Norwegian hospitals was investigated in 2001 [14], 2008 [15] and 2013 [16] demonstrating a steady increase in these services from 27% in 2001, 510% in 2008, and 64% in 2013. In the current study, we found that 54% of hospitals offer CAM, indicating a decrease. However, this decline is smaller compared to the reduction in visits to CAM providers outside of hospital settings. During the same period, the prevalence of such visits dropped from 37% to 21% [1, 13]. The modalities most offered across these years (2001–2019) were acupuncture, expressive arts therapy, and massage.

Our findings of 54% of Norwegian hospitals offering CAM are in line with other studies conducted in Scandinavia. A Danish study [15] reported that 33% of somatic hospitals and 23.1% of psychiatric hospitals offered CAM. A Swedish study [18] found that 62% of psychiatric hospitals offered these modalities. CAM services included mindfulness, basal body awareness, massage or tactile stimulation, acupuncture, and yoga, demonstrating mostly the same modalities as reported in this study. A study from the UK [38] investigated which CAM modality patients were willing to include as part of their care at the hospitals. The most preferred modalities were massage (53%), non-herbal supplements (43%), music therapy (34.8%), acupressure (31%), and meditation/imagery techniques (30%).

### Incorporation of CAM

Previous research demonstrated that medical doctors working in hospital settings are more skeptical towards CAM than General Practitioners (GPs) [17, 37]. However, the interest of the healthcare providers was the main reason for implementing CAM in this study. This is in line with a literature review [23] that found that oncology nurses actively promoted CAM, which they found to correspond with their vision of holistic care [39]. This also corresponds with the results from the 2001 and 2008 Norwegian studies [14, 15]. These studies reported that the incorporation of acupuncture was driven by leadership, which aligns with the findings from this study. Acupuncture has been a part of clinical practice for many Norwegian GPs [40]. This study found that 60% used acupuncture to treat patients. The modality was mostly used to treat musculoskeletal pain, migraine, and tension headaches. The reason for GPs’ positive attitude to acupuncture may be that they endorse CAM modalities that are the closest to conventional medicine [41].

### Reasons for implementing CAM

This study reported that scientific evidence for effect was one reason for implementing CAM at the hospital, which is in line with findings from the 2008 Norwegian study [15]. The perceived lack of evidence for effect was also a reason for not introducing CAM at the hospitals. This is in line with other studies [23, 25]. One study [25] investigated what knowledge different healthcare providers (conventional and CAM) have to guide cancer patients about risks associated with CAM when combined with conventional cancer treatment. The majority (89%) of medical doctors and nurses believed that CAM should be subjected to more scientific testing before being accepted by conventional healthcare providers.

Another reason for implementing CAM was the perception among healthcare providers that conventional medicine does not work for everyone. This was especially true for children and people suffering from psychosis. This aligns with a study investigating CAM use in childhood cancer reporting that children should not be treated as small adults [42]. A review [43] that investigated art therapy for people with psychosis found inconclusive evidence for the effectiveness of art therapy in adults with psychosis, based on highly qualitative articles. However, high-quality qualitative studies indicated that therapists and clients considered art therapy a beneficial, meaningful, and acceptable intervention, although this conclusion was based on few studies.

Holism is a treatment philosophy that applies *wholeness* as the principle of reason as opposed to the study of isolated parts [44]. The participants in this study believed that CAM is a holistic approach and that these modalities are needed in hospitals because people are different and have different health needs. The results of this survey support this perception, showing that somatic and substance-abuse hospitals tend to offer more hands-on treatments, such as acupuncture and massage, whereas psychiatric hospitals and rehabilitation clinics focus on more holistic approaches, including yoga, meditation, and expressive arts therapies. This complements another Norwegian study [45], where the therapists at an outpatient clinic for severely traumatized patients applied holistic treatment approaches based on their understanding of mind and body as one entity.

### Patient safety

The healthcare participants in this study believed that CAM is safe. This contrasts with a Norwegian study [25] which reported that 94% of medical doctors and 93% of nurses agreed that CAM can cause adverse effects. They also believed that it is risky to combine CAM and conventional medicine (78% and 93% respectively). The observed discrepancy between the perspectives of healthcare providers in these studies may stem from differing beliefs. CAM providers often consider CAM to be *natural* and, therefore, associate it with lower risk. Furthermore, healthcare providers, such as nurses, who emphasize holistic care may hold more favorable attitudes toward CAM [39]. This could explain the more positive perceptions of CAM among participants in the current study compared to those in the Norwegian study [25], which focused on oncology healthcare providers working in somatic hospitals.

### Methodology strength and limitations

The response rate of 74% was notably high, aligning closely with the rate of 74% in the 2013 study [16]. However, it was somewhat lower than the rates observed in the 2001 (94%) and 2008 (85%) studies. This high response rate may be attributed to the short and straightforward questionnaire used in this study, which required only one minute for the senior medical officer to complete.

The incorporation of CAM into the official healthcare system is influenced by varying attitudes, beliefs, and perceptions among healthcare providers. Consequently, it is possible that hospitals with a more favorable view of CAM were more likely to respond than those that were more skeptical of these services.

This study relied on the senior medical advisor’s understanding of CAM (although a list of 43 modalities was provided) and their overview of CAM activities within the hospital. Additionally, most healthcare providers preferred not to be associated with CAM, and some participants disagreed with the fact that they practiced CAM. Consistent with this, none of the participants had formal job descriptions related to CAM roles. These factors may contribute to an underestimation of CAM services provided. Moreover, several medical officers in the hospitals gave additional information on CAM offered in other departments within the hospital. This reinforces the suspicion that the extent of CAM offered could have been underestimated, meaning that our total estimate of CAM offered may have been slightly underestimated.

### Implication for practice

Since more than half of Norwegian hospitals offer some form of CAM without a formal job description, there is a clear need for practical guidance on its implementation. Additionally, implementation studies are necessary to identify and understand the barriers and facilitators involved in implementing CAM into healthcare settings. Additionally, Norwegian hospital providers require targeted information and knowledge regarding the possibilities and limitations of CAM for patients [23, 46]. This need has been highlighted by the World Health Organization (WHO) and Norway’s public reports on alternative medicine (CAM) from 1998 [37, 47]. To bridge this gap, it is essential to offer (online) courses that enhance healthcare providers’ understanding of CAM. Conducting research in this field is also crucial to offer patients the best possible evidence-based support when they choose to combine conventional medicine with CAM for their health concerns.

## Conclusion

This study demonstrates that CAM is widely offered in Norwegian hospitals, consistent with previous research. While participants in the process evaluation viewed CAM as a holistic and beneficial approach to care, its adoption remains inconsistent across institutions. This variability, likely due to the lack of defined CAM roles and job descriptions, may pose indirect risks to both healthcare providers and patients. Standardized guidelines are needed to ensure safe and fair implementation.

## Supplementary Information

Supplementary Material 1.

Supplementary Material 2.

## Data Availability

The dataset underlying this paper has not been deposited in any specific repository. However, all datasets and materials used in this study are available upon reasonable request from the corresponding author. Interested parties seeking access to the data must be willing to comply with Norwegian privacy regulations, ensuring adherence to stringent data protection standards.

## Abbreviations

NAFKAM: National Research Center of Complementary and Alternative Medicine
CAM: Complementary and Alternative Medicine
NA: Not Applicable
RHAs: Regional Health Authorities
UiT: The Arctic University of Norway
TSB: Substance Abuse Treatment
BUP: Child and Adolescent Psychiatric Outpatient Clinic
BUPA: Child and Adolescent Psychiatric Clinic
DPS: District Psychiatric Center
EMDR: Eye Movement Desensitization and Reprocessing
CBT: Cognitive Behavioral Therapy
SPSS: Statistical Package for the Social Sciences
WHO: World Health Organization
SIKT: Norwegian Knowledge Sector’s Service Provider
