# Supplementary material for: Investigating complementary and alternative medicine in Norwegian hospitals: a cross-sectional study with process evaluation
Source: BMC Complement Med Ther. 2026 Mar 11;26:192. doi: 10.1186/s12906-026-05339-w (PMC13188417; doi:10.1186/s12906-026-05339-w)
Supplement: Supplementary file 1 — Supplementary Material 1. [file 12906_2026_5339_MOESM1_ESM.pdf]

Intervjuguide: Bruk av alternativ behandling på norske sykehus

Aim: Explore how CAM is practiced in Norwegian hospitals

Present me and my background

Background information about the participants

Gender /Age /Work title

Conventional healthcare profession (nurse, GP)

CAM provider

Year in clinical practice as a conventional professional

(Previous work; Present work)

Name of workplace/hospital

Year in clinical practice as a CAM provider

Conventional training

Tell me about your conventional healthcare training

Is your training relevant to the work you do at the hospital / how?

Do you need more training to give safe and competent treatment?

Do you get someone to replace you if you are on leave/ vacation/ or when you retired

Do you have a work prescription for your CAM position at the hospital?

CAM training and practice

Tell me about your CAM training

What kind of CAM modality do you offer at the hospital/

Tell me about the implementation process/ How did this offer come about?

What do you think about this offer?

How is this treatment carried out in practice?

In your opinion, how do patients experience the treatment? Advantages and disadvantages

In your opinion, why is it important to offer CAM in your department?

What does CAM provide to patients that conventional medical treatment does not?

If you offer one or more CAM treatments, what determines which treatments you provide?

Collaboration:

Do you collaborate with other professionals at the hospital regarding patients?

How does this collaboration take place? (Referring patients). Give examples.

<u>Patients</u>

Who is offered CAM at the hospital? Are there any criteria?

<u>Adverse effects of treatment</u>

Have you experienced anyone getting adverse effects from CAM? Please elaborate

<u>Conclusion</u>

Anything else you would like to add?

Sum up
