# Supplementary material for: Investigating complementary and alternative medicine in Norwegian hospitals: a cross-sectional study with process evaluation
Source: BMC Complement Med Ther. 2026 Mar 11;26:192. doi: 10.1186/s12906-026-05339-w (PMC13188417; doi:10.1186/s12906-026-05339-w)
Supplement: Supplementary file 2 — Supplementary Material 2. [file 12906_2026_5339_MOESM2_ESM.pdf]

Use of CAM: ______________________(hospital)

CAM offered at our hospital:

Acupuncture ☐
Senior medical officer: ________________________________

Massage therapy ☐
Senior medical officer: ________________________________

Psychotherapy (excluding services by psychologists/psychiatrists) ☐
Senior medical officer:________________________________

Expressive art therapy: ☐
Senior medical officer: ________________________________

Diet recommendations: ☐
Senior medical officer________________________________

Other CAM modalities: ☐

Please spesify: ____________________________________

Senior medical officer: ________________________________

No CAM offered at the hospital ☐

Please return this for to:

Nasjonalt forskningssenter innen komplementær og alternativ medisin
UiT Norges arktiske universitet
9037 TROMSØ

E-post: solveig.johansson@uit.no

Overview of the most commonly used CAM modalities in alphabetical order

- Acupressure
- Acupuncture
- Alexander Technique
- Alternative Diet
- Aromatherapy
- Art and Expressive Therapy
- Bach Flower Remedies
- Biopathy
- Bioresonance Therapy
- Breathing Therapy
- Ear Acupuncture
- Emotional Freedom Technique (EFT)
- Energy Balancing
- Nutrition Therapy
- Gestalt Therapy
- Healing
- Herbal Medicine
- Homeopathy
- Hypnotherapy
- Kinesiology
- Cupping
- Craniosacral Therapy
- Art and Expressive Therapy
- Sound and Music Therapy
- Magnetic Field Therapy
- Massage
- Meditation
- Mistletoe
- Naprapathy
- Naturopathy
- Nature Therapy
- Neuro-Linguistic Programming (NLP)
- Osteopathy
- Psychodrama
- Psychosynthesis
- Qigong
- Reflexology
- Rosen Method
- Tai Chi
- Thought Field Therapy (TFT)
- Therapeutic Touch
- Vitamin and Mineral Therapy
- Yoga

The list is not complete
